# Taxifolin as a Major Bioactive Compound in the Vasorelaxant Effect of Different Pigmented Rice Bran Extracts

**DOI:** 10.3389/fphar.2022.799064

**Published:** 2022-03-21

**Authors:** Eun-Hee Seong, Dal-Seong Gong, Saugat Shiwakoti, Deepak Adhikari, Hyun Jung Kim, Min-Ho Oak

**Affiliations:** College of Pharmacy, Mokpo National University, Mokpo, South Korea

**Keywords:** rice bran, taxifolin, cardiovascular diseases, vasorelaxation, eNOS

## Abstract

Cardiovascular disease is one of the leading causes of morbidity and mortality in recent years. The intake of polyphenol rich diets has been associated with improved cardiovascular function and reduced cardiovascular risks. *Oryza sativa* L. is one of the most common cereals worldwide. Rice bran, a byproduct of the rice milling process, contains many bioactive ingredients, including polyphenols, polysaccharides, proteins, and micronutrients. It is also consumed as a healthy diet in the form of rice bran oil and powder in many Asian countries like Japan, South Korea, and India for its several health benefits as a natural antioxidant. Thus, this study evaluated the vasorelaxant effect of ethanolic extracts of brown, green, red, and black rice bran and investigated its underlying vasorelaxant mechanism. Among the four rice bran extracts (RBEs) examined, the red rice bran extract (RRBE) had a strong endothelium-dependent vasorelaxant effect, which was markedly prevented by N-ω-nitro-L-arginine [endothelial nitric oxide synthase (eNOS) inhibitor], wortmannin [phosphoinositide-3 kinase (PI3K) inhibitor], and 1H-[1,2,4]oxadiazole[4,3-alpha]quinoxalin-1-one (inhibitor of guanylate cyclase). Likewise, RRBE induced the phosphorylation of eNOS and Src in cultured endothelial cells, thereby stimulating NO formation. Altogether, these findings propose that RRBE induces endothelium-dependent relaxation, involving at least in part, NO-mediated signaling through the PI3K/eNOS pathway. Further, LC-PDA analysis conducted on the four RBEs also revealed that RRBE highly contained taxifolin, which is an active flavanonol that induces endothelium-dependent vasorelaxation, compared to other RBEs. Subsequently, the underlying mechanism of taxifolin was assessed through vascular reactivity studies with pharmacological inhibitors similar to that of RRBE. These findings deciphered a distinct difference in vasorelaxant effects between RRBE and the other RBEs. We also observed that RRBE induced a potent endothelium-dependent NO-mediated relaxation in coronary artery rings, which involved the Src/PI3K pathway that activates eNOS. Additionally, taxifolin exhibited, at least in part, similar vasoprotective effects of RRBE. Therefore, we propose that RRBE may serve as natural sources of functional phytochemicals that improve cardiovascular diseases associated with disturbed NO production and endothelial dysfunction.

## Introduction

Cardiovascular diseases have become the major cause of worldwide deaths and are a combination of diseases that occur in the heart and major arteries. Approximately 17.9 million people die from cardiovascular diseases (CVD) annually, representing one-third of global mortality (Ali et al., 2021). Research has established that vascular endothelial dysfunction is an important risk factor for developing several cardiovascular diseases including hypertension and atherosclerosis (Landmesser and Drexler, 2005). The endothelium plays a crucial role in balancing vasodilation and vasoconstriction responses through synthesis and release of various endothelium-derived relaxing factors like prostacyclin (prostaglandin I_2_, PGI_2_), nitric oxide (NO), endothelium-dependent hyperpolarization factor (EDHF), and endothelium-derived contracting factors, such as thromboxane A_2_, endothelin, etc. Moreover, endothelial dysfunction is characterized by an imbalance between vasodilation and vasoconstriction substances. This imbalance leads to the impairment of endothelium-dependent relaxation, which is proposed to be the functional characteristic of endothelial dysfunction (Godo and Shimokawa, 2017; Oak et al., 2018).

Nevertheless, numerous studies have reported that regular intake of plant-derived products, such as tea and red wine, reduces the risk of cardiovascular diseases (Khan and Mukhtar, 2018; Castaldo et al., 2019). As reported, these products contain various polyphenols, which protect the vascular system *via* inhibition of endothelial dysfunction and induction of endothelium-dependent vascular relaxation (Yamagata, 2019).

Rice (*Oryza sativa* L.) is one of the most consumed cereals in the world. Rice bran, a byproduct of the rice milling process, is the hard outer layer of rice consisting of aleurone, nucellus, seed coat, and pericarp fractions. However, rice bran is unconsumed due to its instability and difficulty to filter out impurities (Trucksess et al., 2011). Nevertheless, many studies have recently suggested that polyphenols, such as phenolic acid, anthocyanin, and proanthocyanidin, are major antioxidants in rice (Min et al., 2011). Plant polyphenols have been shown to improve vascular function by directly acting as an antioxidant in interacting with reactive oxygen species like superoxide, hydroxy, and peroxy radicals or by activation of endothelial NO synthase (eNOS) through phosphoinositide-3 kinase (PI3K)/Akt pathway (Oak et al., 2018; Shiwakoti et al., 2020). Thus, with increasing interest in health worldwide, various pigmented rice including black, red, brown, and green, is being consumed for its natural antioxidant properties. Furthermore, while non-pigmented rice contains phenolic acid, pigmented rice is rich in polyphenols, such as anthocyanin and proanthocyanidin. So, studies have reported these rice types have high utilization value, such as anti-inflammatory, antioxidant (Callcott et al., 2019b; Callcott et al., 2019a), and cardioprotective (Xia et al., 2003) effects. Thus, the cardiovascular protective effect of rice brans may be attributed to their antioxidant activity. Alternatively, rice bran has the highest total phenolic acid content of the four fractions of whole rice grains. It also contains various bioactive ingredients, including oils, polysaccharides, proteins, and micronutrients (Park et al., 2017). Many studies have therefore indicated that rice bran has several beneficial properties, such as antioxidant (Adebiyi et al., 2008), antitumor (Wang et al., 2016), anti-inflammatory (Kurtys et al., 2018), and antihypertensive (Shobako et al., 2019) effects.

However, until now, no study comparing the cardiovascular protective effects of various types of pigmented rice bran, including their vasodilatory effects, has been conducted. In addition, the derivation of active ingredients from this product also remains unclear. Therefore, this study evaluated the vasorelaxant effect of different pigmented rice brans in the porcine’s coronary artery, elucidated the active compound responsible for this effect, and investigated its underlying mechanism of action.

## Materials and Methods

### Plant Material

Four rice (*Oryza sativa* L.) cultivars of different bran colors [brown, HYUNMI; red, JEOKMI; black, HEUKHYANGMI; green, NOKMI] were harvested in 2017 from Wooriwon Inc. (Boseong, Korea). Then, the Ministry of Agriculture, Food, and Rural Affairs certified these cultivars as organic products. Subsequently, a voucher specimen was deposited at the College of Pharmacy, Mokpo National University’s herbarium (No. P2017RB001-004).

### Preparation and Extraction of Rice Bran

To obtain milled rice brans, all paddy rice samples were dehulled and polished using rice dehuskers and a rice milling machine (FS-2000, Focus Co. Ltd., South Korea), set at 92% of milling ratio to produce 8% of bran from total rice content. Then, to separate grains from the rice bran, these grains were sieved through a 150 μm sieve. Next, all bran samples were extracted thrice with 70% v/v ethanol at 1:10 (w/v) bran-to-solvent ratio for 2 h at 80°C by a heating mantle system connected to a digital feedback temperature controller (WHM-C10D, Daihan Scientific Co. Ltd., Korea), after which the liquid extracts, filtrated with filter paper (Advantec No. 2, Toyo Roshi Kaisha Ltd., Japan) were evaporated *in vacuo* to obtain crude extracts using a rotary evaporator (N-1200A, Eyela, Japan). Dry yield extracts from 100 g of each brown, red, green, and black rice brans were 9.6, 9.3, 10.2, and 7.5 g, respectively. Finally, each dry extract was stored at −20°C until use. On each experimental day, extracts were reconstituted in dimethyl sulfoxide at a concentration of 100 mg/ml for biological assays.

### Reagents and Chemicals

Cayman Chemical (Ann Arbor, MI, United States) supplied the thromboxane A_2_ analog; U46619. Alternatively, indomethacin, N-ω-nitro-L-arginine (L-NA), bradykinin, soluble guanylate cyclase (sGC) inhibitor (1H-[1,2,4]oxadiazolo[4,3-alpha]quinoxalin-1-one, ODQ), wortmannin, and taxifolin were obtained from Sigma-Aldrich (St. Louis, MO, United States). All other chemicals were of analytical grade. HPLC grade solvents (Fisher Scientific) were used for all HPLC-hyphenated experiments. Also, extra pure-grade solvents were used for extraction.

### Tissue Preparation

All tissues used in this study were acquired postmortem from a commercial slaughterhouse. Therefore, they were not subject to institutional animal protocol approval. The slaughterhouse procedures followed the USDA and Humane Slaughter Act guidelines for the care and slaughter of swine. Right after sacrifice, pig hearts were collected from a local slaughterhouse (Mokpo, Jeonnam, South Korea) and moved to the laboratory within 20 min in Krebs bicarbonate solution (119 mM NaCl, 4.7 mM KCl, 1.18 mM KH_2_PO_4_, 1.18 mM MgSO_4_, 1.25 mM CaCl_2_, 1.25 mM NaHCO_3_, and 11 mM D-glucose; pH 7.4) at 4°C. Porcine left coronary artery was isolated immediately upon arriving at the laboratory. The left anterior descending coronary artery was dissected free of fat and connective tissue in oxygenated (95% O_2_ and 5% CO_2_) Krebs bicarbonate solution and cut into 3–4 mm rings. The rings were then mounted on two stainless steel hooks in 10 ml organ baths. One of these hooks was attached to a force transducer to measure changes in isometric tension and coupled to an amplifier and a computer for data collection. In experiments requiring endothelium-denuded rings, the endothelium was removed by rubbing the intimal surface with a pair of forceps. Rings were allowed to equilibrate for at least 60 min at a resting tension of 5 g, a period during which the tension was adjusted and the bathing solution was periodically changed every 15 min.

### Vascular Reactivity Study

The vascular reactivity study was conducted as described previously (Sharma et al., 2019). Briefly, following equilibration, the viability of each coronary ring was determined by repetitive contraction with 80 mM KCl (maximal contraction). Changes in tension caused by the tested concentrations were detected by Grass FT03 force transducers. After a 30 min washout period, rings were contracted with the thromboxane mimetic U46619 (1–60 nM) to about 80% of the maximal contraction and then relaxed with bradykinin (0.3 μM) to check the presence of a functional endothelium as previously reported (Alamgeer et al., 2016). After removing these drugs by repeated washings for three times, it was contracted again with U46619 before constructing a concentration-relaxation curve to rice bran extracts (RBEs) or taxifolin. The interval of test compounds addition is 10 min. In further experiments, rings were also exposed to inhibitors: Nitric oxide(NO) synthase inhibitor [(L-NA, 100 μM)], cyclooxygenase inhibitor (indomethacin, 10 μM), non-selective potassium channel blocker [tetraethylammonium (TEA, 1 mM)], sGC inhibitor (ODQ, 10 μM) and PI3K inhibitor (wortmannin, 30 nM) for 30 min before adding U46619 as performed previously (Panth et al., 2018). In another set of experiments, cumulative contractile responses induced by U46619 (1–300 nM) were obtained in the presence of red rice bran extract (RRBE).

### Culture of Porcine Coronary Artery Endothelial Cells

Segments of porcine coronary arteries were washed with PBS without calcium and endothelial cells (ECs) were isolated by incubation with collagenase treatment (type I, Worthington, 1 mg/ml for 15 min at 37°C). Isolated ECs were cultured in MCDB 131 medium (Gibco) supplemented with 10% fetal calf serum, penicillin (100 U/ml), streptomycin (100 U/ml), fungizone (250 μg/ml) and L-glutamine (2 mM). All experiments were performed with confluent cultures of cells at the first passage.

### Western Blotting

Porcine coronary artery endothelial cells were washed with ice-cold PBS and lysed with a RIPA buffer. Equal protein concentrations (10 μg/lane) were then separated on a denaturing sodium dodecyl sulfate (SDS)-polyacrylamide gel. Subsequently, separated proteins were transferred to a polyvinylidene difluoride (PVDF) membrane at 100 V for 2 h. Next, the membranes were blocked at room temperature in TBS using bovine serum albumin in 0.1% Tween 20 for 1 h. To detect proteins, membranes were incubated with the respective primary antibody: eNOS phosphorylated at Ser1127 [p-eNOS (Ser1177)] (1:1000, Cell Signaling Technology, MA, United States), Src phosphorylated at Tyr416 [p-Src (Tyr416)] (1:1000, Cell Signaling Technology, MA, United States), and β-tubulin (1:1000, Cell Signaling Technology, MA, United States) overnight at 4°C. After washing, membranes were further incubated with the appropriate horseradish peroxidase-conjugated anti-mouse or anti-rabbit immunoglobulin G (1:2000, Cell Signaling Technology, MA, United States) and developed using an enhanced chemiluminescence (ECL) detection kit. Finally, band densities were determined using the ImageJ software. Expression levels of these target proteins were analyzed in at least three individual experiments.

### LC-PDA and LC-MS Analyses

HPLC was conducted using a Waters HPLC system (Waters Corporation, Milford, MA, United States). This system was composed of a 1525 binary pump, coupled with a column oven, 2707 autosampler, and a 2998 photodiode array detector (210–400 nm) attached to a SunFire^®^ C18 column (5 μm, 4.6 × 150 mm, Waters). The linear gradient system was adopted to elute previously extracted rice bran extracts (RBEs) and taxifolin using acetonitrile and water (0.1% formic acid), i.e., from 10% (0 min) to 40% acetonitrile (40 min), followed by an isocratic solvent 100% acetonitrile (10 min) at a flow rate of 1.0 ml/min. Then, the UV absorption was monitored and recorded at 280 nm. For HPLC quantification of taxifolin in RRBE, a stock solution of taxifolin was prepared (1000 μg/ml) and serially diluted with methanol to make standard solutions that ranged from 1 to 300 μg/ml. Subsequent HPLC analyses were conducted under the same gradient solvent condition described above.

Alternatively, LC-MS experiments were conducted on an Agilent 6120 single quadruple MS coupled to an Agilent 1260 Infinity series (Agilent Technologies, Santa Clara, CA, United States), using a SunFire^®^ C18 column (3.5 μm, 3.0 × 100 mm, Waters). The gradient solvent system with acetonitrile and water (0.1% formic acid), 10–40% acetonitrile for 20 min at 0.5 ml/min. MS detection was then used to conduct an electrospray ionization (ESI) with an API source. Finally, MS spectra were obtained between *m*/*z* 100–1000 in negative mode (gas temperature: 350°C, drying gas: 12.0–13.0 L/min, nebulizer pressure: 35–60 psi, quadrupole temperature: 350–500°C, and capillary voltage: 3000 V).

### The Determination of Total Phenolic and Flavonoid Contents

Total phenolic contents were measured based on a previously reported high-throughput microplate assay (Herald et al., 2012). The extract was prepared at concentration of 0.1, 1, 10 mg/ml. For this analysis, a diluted extract (50 μl) was mixed with freshly prepared Folin-Ciocalteu reagent [250 μl, 1:10 (v/v) in water] (Sigma-Aldrich, St. Louis, MO, United States). After 6 min, 100 μl Na_2_CO_3_ (75 mg/ml) was added to each well and left in the dark for 90 min. The absorbance was measured at 765 nm on a spectrophotometric microplate reader (PerkinElmer, MA, United States). Gallic acid (0.01 mg/ml to 0.05 mg/ml) was used as a standard to prepare the calibration curve (*R* ^2^ = 0.9995), after which the total phenolic content was expressed as μg of gallic acid equivalents per g of sample. The total flavonoid content was determined using the aluminium chloride (AlCl_3_) spectrophotometric method as previously described (Chang et al., 2002). For the analysis, 30 μl of the sample solution (0.1, 1, 10 mg/ml) was mixed with methanol (90 μl) and distilled water (170 μl). The mixture was then added with 6 μl of 10% w/v AlCl_3_ and 1 M potassium acetate. Mixed solutions were subsequently left in the dark for 30 min, after which the absorbance was measured at 415 nm using a microplate reader. Quercetin (10–100 μg/ml) was used as a standard to prepare the calibration curve (*R* ^2^ = 0.9983). Afterward, the total flavonoid content was calculated following the standard calibration curve, expressed as μg of quercetin equivalents per g of sample. Each test solution was analyzed in triplicate.

### Statistical Analysis

Data are presented as mean values ± standard error of the mean (SEM) for *n* animals (one coronary artery ring per animal for each treatment). One-way analysis of variance was used to define statistically significant differences between groups, followed by Bonferroni’s *post-hoc* test. The Prism software (GraphPad Inc., La Jolla, CA, United States) was used for all analyses. A *p*-value < 0.05 was considered significant.

## Results

### Comparison of Vasorelaxant Effects of Different Pigmented RBEs

**FIGURE 1:**
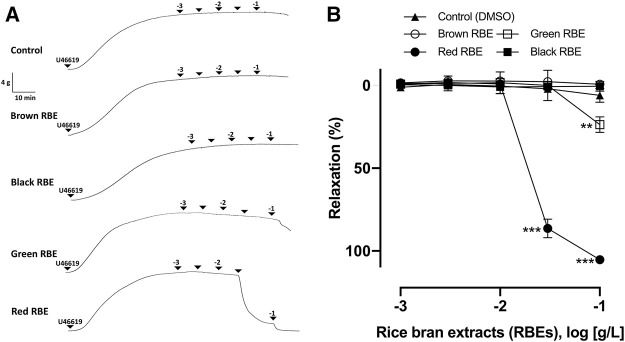
Characterization of vasorelaxation in four pigmented RBEs in porcine coronary artery rings. Arterial rings with the endothelium were contracted with U46619 before the addition of increasing concentrations of red, brown, green, and black RBEs (1 μg/ml to 100 μg/ml) to construct the concentration-relaxation curves. **(A)** Representative original tracing and **(B)** corresponding cumulative concentration-relaxation curve. The relaxation response is expressed as the percentage relaxation of the U46619-induced contraction. Results are presented as means ± SEM (*n* = 6–8). ***p < 0.001 *vs* control. **p < 0.01 *vs* control.

Four dried pigmented rice bran types, including black, brown, red, and green rice brans, were milled. They were then extracted with 70% ethanol and prepared as RBEs, respectively. The evaluation of their vasorelaxant effects was conducted following the vascular reactivity study, using porcine coronary artery rings that were pre-contracted with the thromboxane A_2_ receptor agonist, U46619. Among the different pigmented RBEs, RRBE showed the most potent vasorelaxant effect. RRBE induced vascular relaxation in endothelial rings, with an ED_50_ value of 23.50 μg/ml (Figures 1A,B). Results also showed that the relaxation of RRBE started at a concentration greater than 30 μg/ml and reached a near maximal value at 100 μg/ml (E_max_ = 105.24 ± 3.99%), suggesting that the vasorelaxant effects of RRBE were concentration-dependent. In contrast, Green RBE caused vascular relaxation in endothelial rings, with an ED_50_ value of 136.6 μg/ml. As observed with Green RBE, its maximal value of vascular relaxation was 23.65 ± 6.43% at a concentration of 100 μg/ml. However, Black and Brown RBE did not show significant vascular relaxation, even with 100 μg/ml concentration (ED_50_ ≥ 500 μg/ml) treatments.

### The Role of Endothelium in RRBE-Induced Vasorelaxation

**FIGURE 2:**
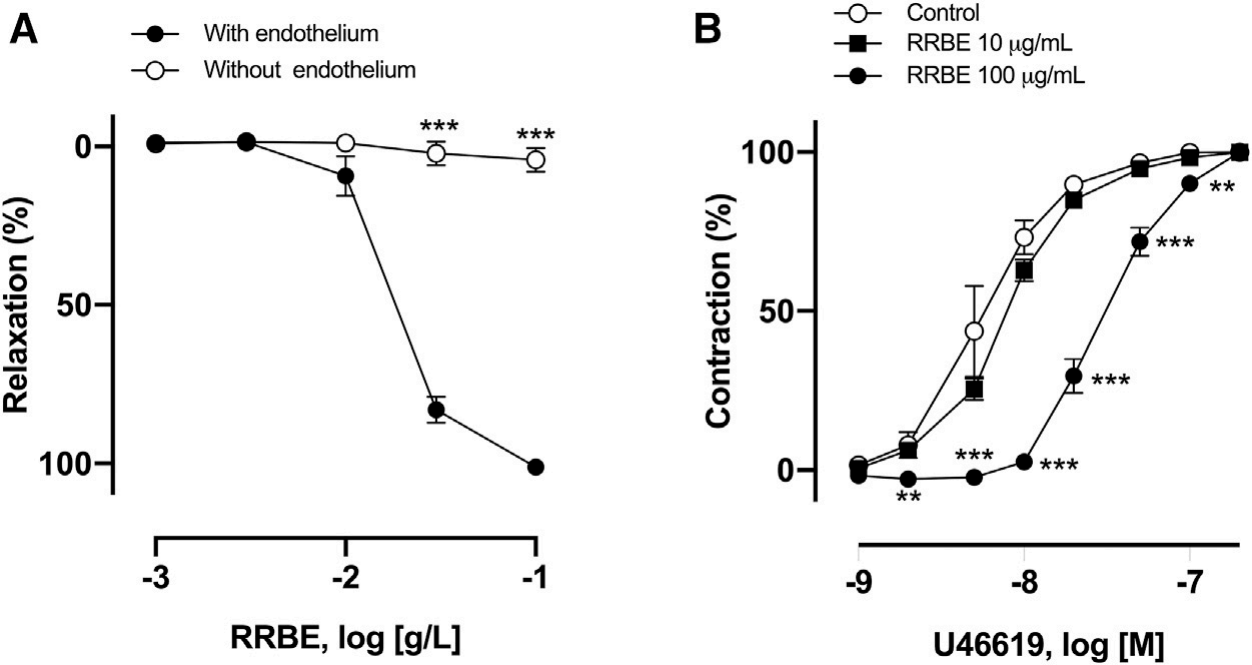
RRBE induced endothelium-dependent relaxation and suppressed contraction in the porcine’s coronary ring. **(A)** Intact and endothelium-denuded rings were contracted using U46619 before the addition of increasing concentrations of RRBE (1 μg/ml to 100 μg/ml) to construct concentration-relaxation curves. ***p < 0.001 *vs* the endothelium. **(B)** Coronary artery rings were treated with RRBE (10 μg/ml and 100 μg/ml) before constructing the concentration–contraction curve in response to U46619. Results are presented as mean ± SEM of five to seven experiments. ***p < 0.001 *vs* control. **p < 0.01 *vs* control.

To investigate the endothelium’s role in RRBE-induced vasorelaxation, concentration responses to RRBE were evaluated in endothelium-denuded rings pre-contracted with U46619. As observed, RRBE induced a concentration-dependent vascular relaxation of the endothelium, but not in the endothelium-denuded coronary artery. RRBE also caused vascular relaxation in rings, with an ED_50_ value of 23.50 μg/ml (E_max_ = 101.15 ± 6.44%) (Figure 2A), suggesting the endothelium-dependent vasorelaxant effect of RRBE.

Subsequently, the possibility that RRBE, besides inducing endothelium-dependent relaxation, also affects vasoconstriction was investigated. The ED_50_ value of vasoconstriction of thromboxane mimetic U46619 alone was 5.5 nM, but in the presence of RRBE (100 μg/ml), the ED_50_ value was increased to 30 nM, suggesting that RRBE prevents U46619-induced vasoconstriction (Figure 2B).

### Characterization of RRBE-Induced Endothelium-Dependent Vasorelaxation

**FIGURE 3:**
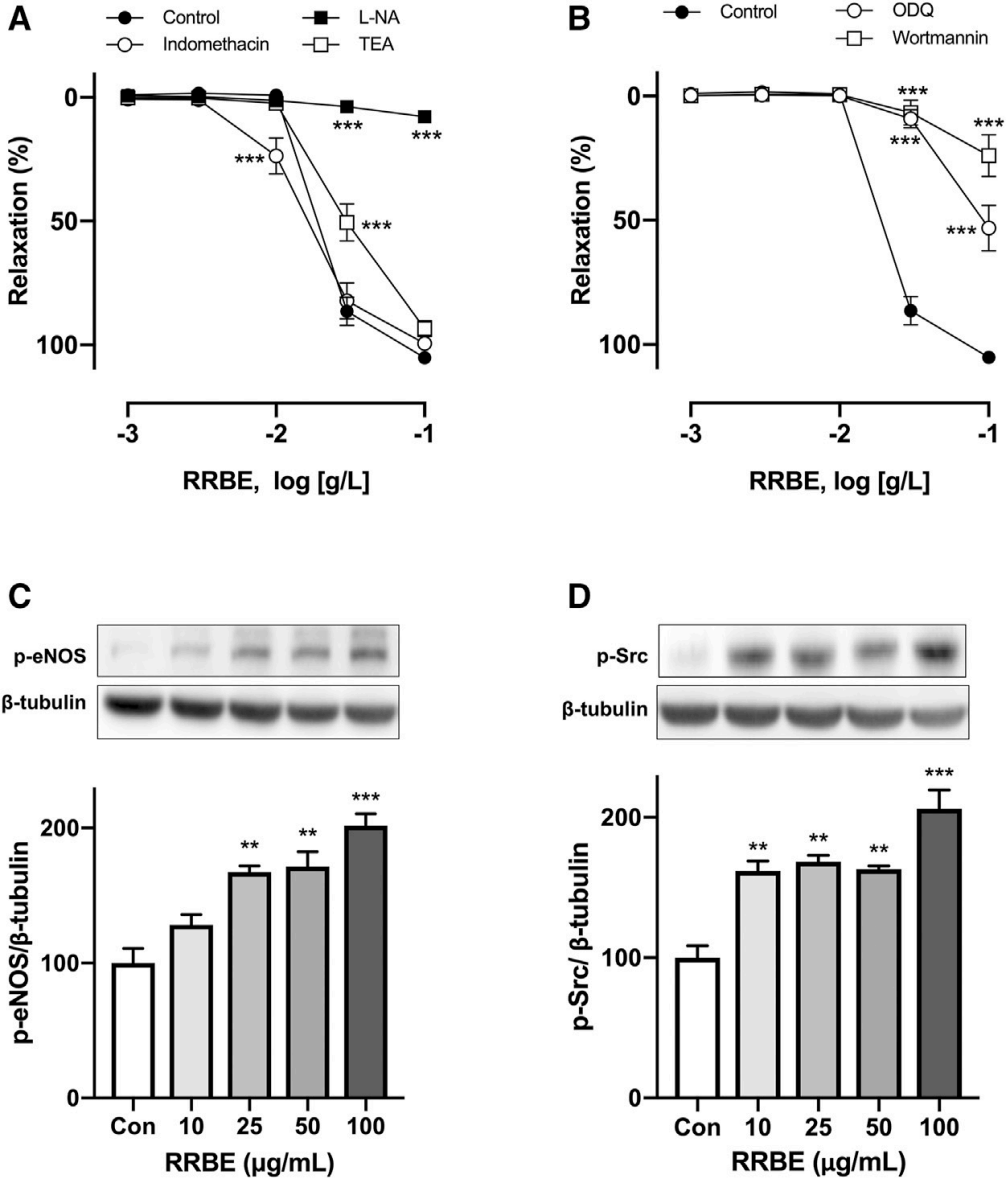
Characterization of endothelium-dependent relaxations in response to RRBE in coronary artery rings. Intact rings were incubated with **(A)** the NO synthase inhibitor (L-NA, 100 μM), cyclooxygenase inhibitor (indomethacin, 10 μM), and non-selective potassium channel blocker (TEA, 1 mM), in addition to **(B)** sGC inhibitor (ODQ, 10 μM) and PI3K inhibitor (wortmannin, 30 nM) for 30 min before the addition of U46619. Results are presented as mean ± SEM of 9–12 experiments. ***p < 0.001 *vs* Control. Representative and cumulative data of phosphorylation level of eNOS at Ser1177 **(C)** and Src at Tyr418 **(D)** in endothelial cells exposed to RRBE as determined by western blot. ***p < 0.001 *vs* control. **p < 0.01 *vs* control.

It is previously reported that three pathways of vascular relaxation exist. These include pathways through the endothelium and NO, PGI_2_, and EDHF (Vanhoutte, 2003). Therefore, to characterize the effect of RRBE on endothelium, vascular reactivity studies were conducted on each pathway in the presence of an inhibitor. In the presence of L-NA (100 μM), an inhibitor of NOS, RRBE-induced vasorelaxations was abolished, with maximum relaxation reduced to 7.82 ± 3.37% at 100 μg/ml (vs Control group 105.24 ± 3.99%, Figure 3A). However, indomethacin, a cyclooxygenase inhibitor (10 μM), and TEA (1 mM), a K^+^ channel blocker, did not affect the maximum relaxation to RRBE with E_max_ values of 99.44 ± 4.47% and 93.42 ± 11.13%, respectively. These results propose that the activation of eNOS plays a major role in RRBE-induced vasorelaxation. Previous studies reported that NO-mediated vasorelaxation was involved in the Src/PI3K/Akt pathway *via* activation of sGC (Feil et al., 2003; Sessa, 2004; Zeller et al., 2009; Thapa et al., 2015). Therefore, subsequent experiments were conducted to determine whether RRBE-induced relaxation was involved with this signal pathway. As observed, the PI3K inhibitor; wortmannin (30 nM), and the inhibitor of guanylate cyclase; ODQ, (10 μM), significantly reduced the endothelium-dependent relaxation of RRBE (Figure 3B, maximal relaxations 23.97 ± 14.89% and 53.12 ± 13.72% to wortmannin and ODQ, respectively). To better characterize the signaling pathway involved in eNOS activation in response to RRBE during NO-mediated relaxation periods, levels of p-Src and p-eNOS were assessed in endothelial cells through immunoblotting. The untreated endothelial cells exhibited either no or only low levels of p-Src and p-eNOS, but RRBE concentration-dependently increased the phosphorylation of Src and eNOS in endothelial cells (Figures 3C,D). Altogether, these findings propose that the RRBE-induced endothelium-dependent vasorelaxation was due to Src/PI3K-mediated NO formation in endothelial cells.

### Determination of Phenolic Acid and Flavonoid Contents of RBEs

Total phenolic and flavonoid contents of the four RBEs were measured using the 96-well plate method (Chang et al., 2002; Herald et al., 2012). The total phenolic acid content was converted to its gallic acid equivalent, whereas the total flavonoid content was converted to its quercetin equivalent (Table 1). Among the four RBEs, RRBE had a total phenolic acid (31.62 ± 1.65 mg GAE/g, DW) and flavonoid contents (3.63 ± 0.08 mg QE/g, DW) compared to other RBE extracts. In particular, RRBE had approximately 3.6 times more total phenolic acid and 2.3 times more flavonoid than green RBE, which has the least phenolic (8.90 ± 0.25 mg GAE/g, DW) and flavonoid (1.61 ± 0.14 mg QE/g, DW) content among four RBE extracts.

**TABLE 1:** Total phenolic and flavonoid contents in different pigmented RBEs.

	Total phenolic content (mg GAE/g, DW)	Total flavonoid content (mg QE/g, DW)
Black RBE	20.52 ± 1.02	1.92 ± 0.36
Brown RBE	12.78 ± 0.31	3.57 ± 0.31
Green RBE	8.90 ± 0.25	1.61 ± 0.14
Red RBE	31.62 ± 1.65	3.63 ± 0.08

Data are reported on a dry weight (DW) rice bran basis and presented as mean ± SEM. GAE, gallic acid equivalent; QE, quercetin equivalent.

### Identification and Quantification of Taxifolin in RRBE

**FIGURE 4:**
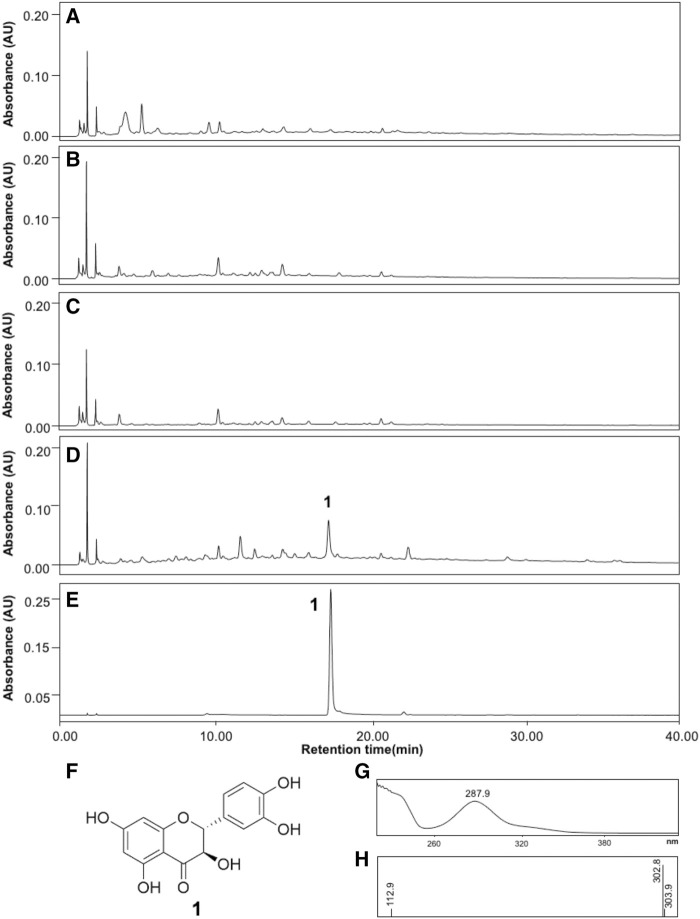
HPLC profiles of the four RBEs and taxifolin. **(A)** Black RBE, **(B)** Brown RBE, **(C)** Green RBE, **(D)** RRBE and, **(E)** Taxifolin. **(F)** Structure of taxifolin, in addition to **(G)** UV and **(H)** MS spectra of the major RRBE and taxifolin peaks recorded in the chromatogram.

To identify active compounds, HPLC analyses of the four RBEs were performed. LC-PDA profiles identified a major peak at tR 17.36 for RRBE, but no major peak was observed in other remaining RBEs (Figures 4A–D). The peak was identified as taxifolin by comparing with previously reported spectral data (Yoon et al., 2020). LC-PDA profiles obtained using a standard taxifolin showed closely similar values at the retention time of 17.40, which was identical to UV spectral patterns of those at a major RRBE peak (Figure 4E). The peak at t_R_ 17.36 in RRBE and taxifolin showed the same UV spectral patterns and maximal UV absorption at 287.9 nm by PDA analysis, indicating an identical structural scaffold, flavanonol type (Figure 4G). Additionally, the MS spectrum of the major RRBE peak showed a molecular ion [M-H]^-^ at *m*/*z* 302.8 (Figure 4H) corresponding to taxifolin (Figure 4F). Likewise, during an HPLC quantification of taxifolin in RRBE, a calibration curve (*R* ^2^ = 0.9994) was produced, using taxifolin as the standard at 1–300 μg/ml. The concentration of taxifolin was then quantified as 225.22 ± 0.89 μg per g of RRBE.

### The Vasorelaxant Activity of Taxifolin and Its Underlying Mechanism

**FIGURE 5:**
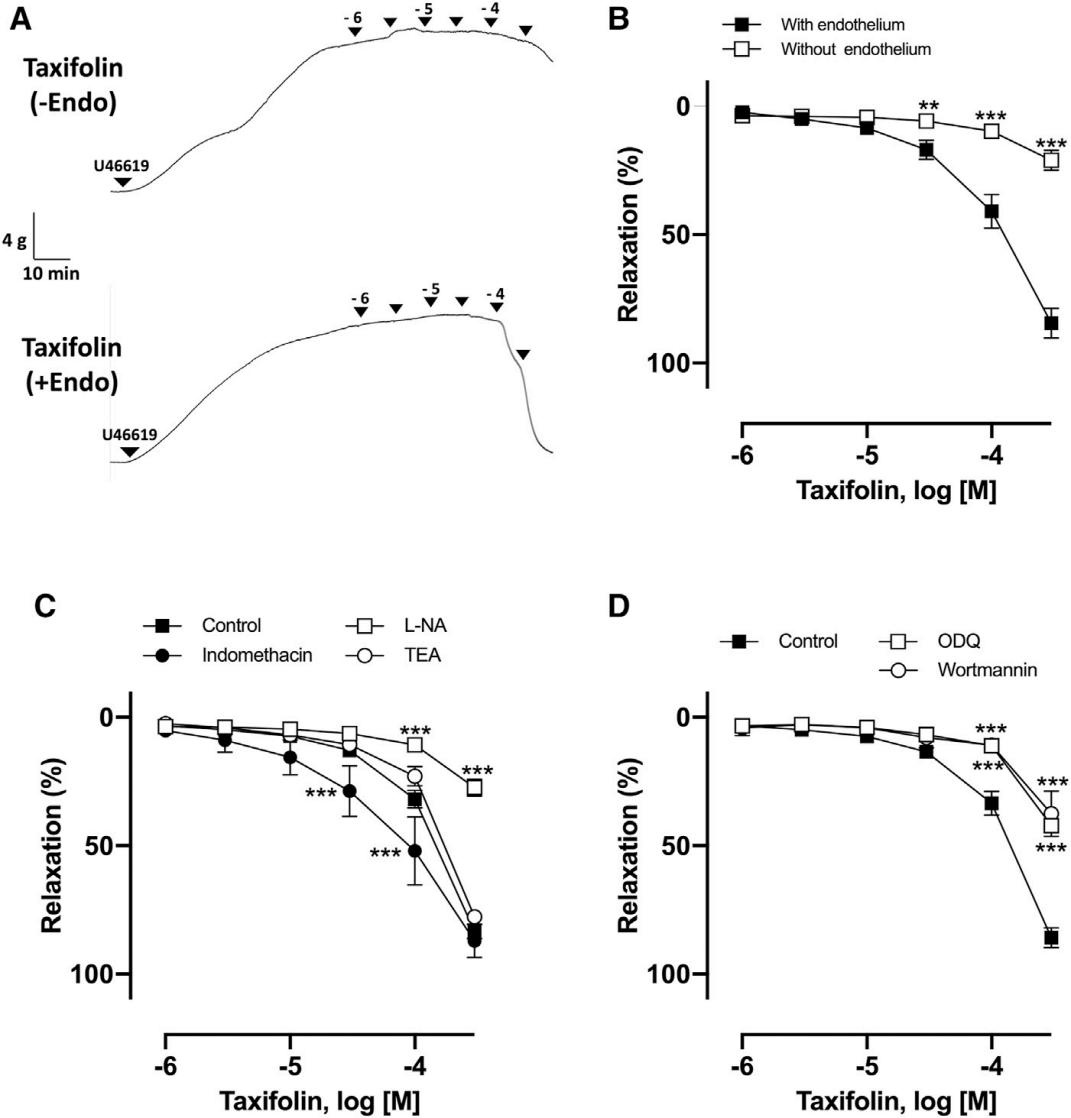
Characterization of endothelium-dependent relaxation to taxifolin in coronary artery rings. **(A)** Representative original tracing of the concentration-relaxation curve. **(B)** Intact and endothelium-denuded rings were contracted using U46619 before the addition of increasing concentrations of taxifolin (1–300 μM) to construct concentration-relaxation curves. ***p < 0.001 vs with endothelium. **p < 0.01 *vs* endothelium. **(C)** Intact rings were incubated with the NO synthase inhibitor (L-NA, 100 μM), cyclooxygenase inhibitor (indomethacin, 10 μM), and non-selective potassium channel blocker (TEA, 1 mM), in addition to **(D)** guanylyl cyclase inhibitor (ODQ, 10 μM) and PI3K inhibitor (wortmannin, 30 nM). Results are presented as mean ± SEM of 8–10 experiments. ***p < 0.001 *vs* control.

The vasorelaxant effect of taxifolin was assessed using porcine coronary artery rings contracted with U46619. As observed, taxifolin concentration-dependently induced relaxation in endothelial rings, with an ED_50_ value of 84.37 ± 24.6 μM (Figures 5A,B). In the presence of L-NA, wortmannin, and ODQ, the vasorelaxant effect of taxifolin was strongly inhibited, with E_max_ values of 30.32 ± 2.54%, 37.60 ± 17.43%, and 42.16 ± 1.91%, respectively, versus taxifolin alone at 100 μM (83.41 ± 6.85%) (Figures 5C,D). Taken together, we propose that taxifolin induced vasorelaxation through the activation of eNOS *via* the PI3K and sGC pathway, suggesting that taxifolin is a major representative of the RRBE’s vasorelaxant effect.

## Discussion

This study demonstrated that RRBE was a potent inducer of endothelium-dependent relaxation in isolated porcine coronary arteries among different pigmented RBEs investigated. We propose that its vasoprotective effect was by eNOS activation *via* the PI3K/eNOS/sGC pathway. Additionally, taxifolin was proposed to represent the major vasoprotective bioactive compound in RRBE.

Rice, one of the most consumed cereals, is derived from the monocot plant (*Oryza sativa* L*.*) and is primarily cultivated in Asian countries, including Korea and Japan (Roy et al., 2011). About 7% of rice bran is produced as a byproduct during the milling process. According to different pigmentation types of the outer layer, whole grain rice can be classified as black, green, red, etc. Pigmented rice is also becoming more popular due to its beneficial cardiovascular effects against atherosclerosis and hypertension, mainly due to its antioxidant properties (Jan-On et al., 2020; Tan et al., 2020). However, comparing the antioxidant properties of different pigmented RBEs, including their vasorelaxant effects and underlying mechanisms, remains unclear (Jun et al., 2012). Thus, in this study, we compared the vasorelaxant effects of different RBEs and found that RRBE showed the most potent endothelium-dependent vasorelaxant effects compared to brown, black, and green RBEs.

The blood vessel is composed of three layers, including an inner membrane consisting of a single layer of endothelial cells, the media containing predominantly smooth muscle cells, elastic fibers, and an outer membrane composed of collagen fibers and nerves in blood vessels. The endothelium plays an essential role in regulating the structure and function of blood vessels, as well as in balancing the vasodilation and contraction response by synthesizing and releasing various vasoconstriction and relaxation factors (Oak et al., 2018). Endothelium-dependent vasodilation responses are primarily mediated by NO, endothelium-dependent hyperpolarization, and PGI_2_, whereas endothelium-dependent vasoconstriction responses are mediated by endothelin-Ⅰ, angiotensin Ⅱ, reactive oxygen species (De Nigris et al., 2005), and thromboxane A_2_. Among them, NO, which is produced through eNOS, is a key factor regulating various responses that contribute to vascular relaxation. In this study, L-NA, which is an endothelial NO synthase inhibitor, markedly inhibited RRBE-induced vasorelaxation. In contrast, the maximal relaxation was not affected by indomethacin, which is an inhibitor of COX and TEA, an inhibitor of the myoendothelial gap junction, ruling out the involvement of vasoactive PGI_2_ and EDHF. Moreover, no vasorelaxant effect of RRBE was observed in artery rings without endothelium. Along with inducing endothelium-dependent vasorelaxation, RRBE significantly blunted contractile responses to vasoconstrictors, such as thromboxane analog, U46619.

It was previously reported that Src/PI3K pathway mediated the stimulatory effects of some plant-derived polyphenols on the endothelial formation of NO, which diffuses into smooth muscle cells and induces a vasodilatory response through the sGC/cGMP/PKG pathway (Feil et al., 2003; Anselm et al., 2007; Kim et al., 2013). Therefore, experiments were conducted to confirm the signaling pathway of RRBE-induced vasorelaxation. Similar to previous studies, our findings showed a rapid and concentration-dependent increase in the phosphorylation of eNOS. Untreated endothelial cells had either no or only a low level of p-Src and p-eNOS, whereas the increase in the concentration of RRBE resulted in an upregulation of p-Src and p-eNOS protein levels. Additionally, wortmannin, an inhibitor of PI3K, which is a major factor for the phosphorylation of p-eNOS, and ODQ, an inhibitor of sGC, was activated in smooth muscle cells by NO, thereby significantly preventing RRBE-dependent vasorelaxation. Altogether, these findings propose that RRBE-induced strong endothelium-dependent relaxations, involving NO-mediated signaling through the PI3K/Akt/eNOS/cGMP pathway.

Epidemiological studies have indicated that regular intake of polyphenol-rich diets, such as red wine and tea, is associated with the prevention of cardiovascular diseases. It has also been proposed that certain polyphenolic-rich products and authentic polyphenols can increase the protective effect of these phytochemicals on the vascular system, especially on the endothelium (Hertog et al., 1993a; Hertog et al., 1993b; Hertog et al., 1997; Oak et al., 2018; Shiwakoti et al., 2020). Indeed, several polyphenol-rich plant and fruit extracts derived from grape, tea, berries, and traditional medicinal plants cause pronounced endothelium-dependent vasorelaxations of pre-contracted arterial rings (Fitzpatrick et al., 1995; Shiwakoti et al., 2020). After confirming the strong vasodilatory effect of RRBE among the different pigmented RBEs evaluated, we hypothesized that observed effects were due to relatively high contents of polyphenol in RRBE compared to others. However, the quantity of polyphenols and flavonoids in pigmented rice bran was not correlated with the potency of their vasorelaxant effects. Furthermore, our results indicated that the quantity of polyphenols in Black RBE was twice greater than that in Green RBE, however, its vasorelaxant effect was similar and even less than that of Green RBE. Additionally, the total polyphenol content of Brown RBE was similar to that of RRBE, however, Brown RBE did not show any significant vasorelaxant effect up to 100 μg/ml. Therefore, HPLC profiling study was conducted to reveal the difference in composition among pigmented RBEs. As a result of comparing their LC-PDA profiles, a noticeable peak that was not observed in other three extracts was detected in RRBE, which was identified as taxifolin by comparison of the previously reported spectral data (Yoon et al., 2020).

Taxifolin (3,5,7,3′,4′-pentahydroxy flavanone or dihydroquercetin) was first isolated from Douglas fir bark [*Pseudotsuga taxifolia* (Lindl.) Britton], and is commonly found in well-known health beneficial plants, including milk thistle, onions, and French maritime pine bark (Sunil and Xu, 2019). In addition, taxifolin was also identified in black rice bran (Sriseadka et al., 2012). It was previously reported that taxifolin showed antioxidant (Guo et al., 2015), lipid-lowering (Casaschi et al., 2004), anti-cancer (Das et al., 2021), and numerous neuroprotective (Saito et al., 2017; Inoue et al., 2019) effects. In addition, taxifolin improved cerebral blood flow and decreased blood pressure *in vivo* model (Plotnikov et al., 2017; Saito et al., 2017). However, the direct vasorelaxant effects of taxifolin and its underlying mechanisms, revealing how it affects the blood vessels remains unclear. Similar to the RRBE, taxifolin concentration-dependently elicited the relaxation of coronary artery rings. Additionally, in the presence of L-NA, an inhibitor of eNOS, the vasorelaxant effects of taxifolin were strongly prevented, but not by indomethacin and TEA. These results propose that taxifolin induced vasorelaxation *via* the formation of NO in the endothelium. Moreover, wortmannin, which is an inhibitor of PI3K, and ODQ, an inhibitor of sGC, significantly inhibited vasorelaxation, which was induced by taxifolin. Altogether, these findings propose that taxifolin induced endothelium-dependent relaxation, involving NO-mediated signaling through the PI3K/Akt/eNOS/cGMP pathway. According to our study, vasorelaxation induced by RRBE alone was higher (E_max_ value of 105.24 ± 3.99%) than taxifolin alone (E_max_ value of 84.44 ± 12.58%). Therefore, the vasorelaxation effect of RRBE may not be only due to taxifolin. Nevertheless, it can be concluded that taxifolin was one of the active compounds in RRBE that accounted for the observed vasorelaxant effect, which was then, most likely, synergically induced by other polyphenol compounds.

Nowadays, various new pigmented rice varieties have been developed because of their abundant phytonutrients and health benefits. Through plant breeding and plant molecular biology techniques, plant breeders have focused on developing new rice varieties with high antioxidant properties that can be used as health-promoting foods (Nam et al., 2006). However, their phenolic and flavonoid contents are significantly different depending on their varieties and environmental conditions during cultivation, such as temperature and humidity (Nagendra Prasad et al., 2011). Therefore, HPLC profiling and polyphenol content in this study did not represent an accurate analytic tool to differentiate all variants of pigmented rice worldwide. So, investigations of different kinds of pigmented rice bran, comparing its effects and composition should be continued.

In conclusion, these findings indicated that among various pigmented rice bran, RRBE induced potent endothelium-dependent NO-mediated relaxation in coronary artery rings, which involved the Src/PI3K/Akt pathway, leading to the activation of eNOS. Additionally, taxifolin in RRBE accounted for the vasoprotective effects of RRBE. Therefore, RRBE and its extracts are proposed to serve as natural sources of functional phytochemicals for developing various medicinal foods against cardiovascular diseases associated with disturbed NO production and endothelial dysfunction.

## Data Availability

The original contributions presented in the study are included in the article/Supplementary Material, further inquiries can be directed to the corresponding authors.
